# Multisensory cueing facilitates naming in aphasia

**DOI:** 10.1186/s12984-020-00751-w

**Published:** 2020-09-09

**Authors:** Klaudia Grechuta, Belén Rubio Ballester, Rosa Espín Munné, Teresa Usabiaga Bernal, Begoña Molina Hervás, Bettina Mohr, Friedemann Pulvermüller, Rosa Maria San Segundo, Paul F. M. J. Verschure

**Affiliations:** 1 Institute for Bioengineering of Catalonia (IBEC), Av. d’Eduard Maristany 16, 08019 Barcelona, Spain; 2 Servei de Medicina Física i Rehabilitació de l’Hospital Univ. de Tarragona, 43-005 Tarragona, Spain; 3 Charite Universitätsmedizin Berlin, 10-117 Berlin, Germany; 4 Freie University Berlin, Brain Language Laboratory, DPH, WE4, 14-195 Berlin, Germany; 5 Humboldt Universität, BSMB, 10-099 Berlin, Germany; 6 Einstein Center for Neurosciences, 10-117 Berlin, Germany; 7 Institució Catalana de Recerca i Estudis Avançats, 08-010 Barcelona, Spain

**Keywords:** Stroke, Aphasia, Lexical access, Word-finding, Multisensory cueing, Neurorehabilitation

## Abstract

**Background:**

Impaired naming is a ubiquitous symptom in all types of aphasia, which often adversely impacts independence, quality of life, and recovery of affected individuals. Previous research has demonstrated that naming can be facilitated by phonological and semantic cueing strategies that are largely incorporated into the treatment of anomic disturbances. Beneficial effects of cueing, whereby naming becomes faster and more accurate, are often attributed to the priming mechanisms occurring within the distributed language network.

**Objective:**

We proposed and explored two novel cueing techniques: (1) Silent Visuomotor Cues (SVC), which provided articulatory information of target words presented in the form of silent videos, and (2) Semantic Auditory Cues (SAC), which consisted of acoustic information semantically relevant to target words (ringing for “telephone”). Grounded in neurophysiological evidence, we hypothesized that both SVC and SAC might aid communicative effectiveness possibly by triggering activity in perceptual and semantic language regions, respectively.

**Methods:**

Ten participants with chronic non-fluent aphasia were recruited for a longitudinal clinical intervention. Participants were split into dyads (i.e., five pairs of two participants) and required to engage in a turn-based peer-to-peer language game using the Rehabilitation Gaming System for aphasia (RGSa). The objective of the RGSa sessions was to practice communicative acts, such as making a request. We administered SVCs and SACs in a pseudorandomized manner at the moment when the active player selected the object to be requested from the interlocutor. For the analysis, we compared the times from selection to the reception of the desired object between cued and non-cued trials.

**Results:**

Naming accuracy, as measured by a standard clinical scale, significantly improved for all stimuli at each evaluation point, including the follow-up. Moreover, the results yielded beneficial effects of both SVC and SAC cues on word naming, especially at the early intervention sessions when the exposure to the target lexicon was infrequent.

**Conclusions:**

This study supports the efficacy of the proposed cueing strategies which could be integrated into the clinic or mobile technology to aid naming even at the chronic stages of aphasia. These findings are consistent with sensorimotor accounts of language processing, suggesting a coupling between language, motor, and semantic brain regions.

**Trial registration:**

NCT02928822. Registered 30 May 2016.

## Introduction

About 30% of stroke patients worldwide suffer from aphasia, and the majority remains chronic [1]. Anomia, or word-finding difficulty, is a ubiquitous characteristic of aphasia, which significantly compromises communication and quality of life of individuals affected by stroke [2, 3]. Consequently, the rehabilitation of language disorders largely incorporates strategies fostering the recovery of impaired naming and communication by facilitating access to linguistic content.

To map a lexical concept to verbal structure requires multiple steps [4, 5]. First, there is the intention to articulate a specific concept in speech, followed by the so-called lexical access, which consists of the retrieval of a target word from a lexicon [6, 7]. At this stage, the focused concept activates the target lemma, the semantic and syntactic properties of the lexical item [8], triggering the speech form-defining, phonological system. The latter provides verbal execution where the articulatory shape of a word, in the context of other words, forms a sentence-like utterance [9]. In stroke-induced aphasia, depending on the lesion site and extent, some or all stages of this naming process might be impaired, leading to high variability in language performance deficits among affected individuals. Consequently, standard naming therapy, or the so-called cueing, is designed to address different phases and aspects of both retrieval and production [3]. For example, the well-established phonological cueing approach targets the ability to retrieve phonemes underlying the articulation of a word [10, 11]. To this aim, patients are given verbal cues that provide initial sound/s of the target word (e.g., “p” for “pancake”). Another therapeutic method is semantic cueing, which targets the activation of lexical-semantic association networks [12, 13]. As such, semantic cueing consists of providing information that categorizes, describes, or defines target words (e.g., “it goes well with maple syrup” for a pancake).

In the clinical context, cueing is considered beneficial because it facilitates naming, consequently resulting in higher accuracy and faster reaction times of speech production. Indeed, phonological, semantic, and mixed approaches substantially improve not only immediate but also long-term naming performance as well as functional communicative effectiveness [14–18]. Critically, similar effects are reported when the cues are administered through technology-based methods, even to individuals with persisting aphasia [19–23]. This finding is particularly relevant in the context of rapid advancement of self-managed, computer-based exercises for individuals with aphasia, which are becoming widely tested and used not only as a part of the clinical inpatient care during the acute and subacute stages but also after the hospital discharge at patient’s homes [24].

The beneficial effects of cueing, whereby the naming of target words becomes faster and more accurate, are usually attributed to priming mechanisms occurring within residual language network bilaterally [25–27]. Depending on the type of administered cues (e.g., initial phoneme, full word), imaging studies report increased activity in regions including the right anterior insula, inferior frontal, and dorsal anterior cingulate cortices, as well as the left premotor cortex [28]. One account yields that cueing elicits activation of lexical representations at phonological and semantic levels in a selective manner [29], thus enabling the recovery of phonological or semantic deficits, respectively. This hypothesis, however, seems at odds with the notion that during therapeutic tasks such as picture naming semantic information contained by the stimuli might automatically activate phonological information and vice-versa [30, 31]. This interpretation might be explained by the interactive activation approach to word production, which proposes that lexical retrieval occurs within a distributed language network, in which nodes are connected across semantic, lexical, and phonological levels of representation in a feedforward and feedback manner (i.e., bidirectionally) [5]. Indeed, an analysis of the language connectome in both healthy controls and brain tumor patients showed a broad network spanning about 25% of the total human connectome [32]. Following this architecture, therapy-induced stimulation at the level of the semantic system can activate phonological and orthographical processing, and vice-versa. This, in turn, may explain why several studies report higher efficacy of a combined (i.e., mixed) cueing therapy rather than when semantic or phonological primes are delivered independently [18, 30]. Further supporting evidence for this network interpretation is the observation that speech perception is governed by general principles of statistical inference across all available perceptual sources [33] suggesting that similar principles of Bayesian inference are involved in cueing based rehabilitation strategies.

In this study, we aimed to explicitly test whether multisensory signals, processed by more than one sensory channel, and driven by the statistics of real-world interactions [34, 35] aid naming in chronic non-fluent aphasia. Such a finding would further support the role of inference-based networks at the basis of the processing of language and its deficits, as evidenced by previous studies [33, 36]. To this end, we proposed two novel cueing strategies and investigated their effects on naming in the context of a within-subjects longitudinal clinical study with post-stroke aphasia patients. On the one hand, we investigated the so-called Silent Visuomotor Cues (SVC) strategy. SVCs provided articulatory information of target words presented in the form of silent videos which display lip movements of a speech and language therapist during naming [37]. On the other hand, we studied Semantic Auditory Cues (SAC). Here, the primes consisted of acoustic information semantically relevant to target words such as the sound of ringing for “telephone,” or the sound of an engine revving up for “car.”

First, the motivation to investigate SVC was grounded in neurophysiological evidence, which strongly supports the notion of perceptual functions of speech production centers. In particular, it has been demonstrated that part of the ventrolateral frontal cortex in humans (Brodmann’s 44), initially thought to be engaged in the control of motor aspects of speech production exclusively [38, 39], is also involved in the processing of orofacial gestures [40, 41]. This is well illustrated in a MEG (magnetoencephalography) study in which the authors compared the activation of the human Mirror-Neuron System, (MNS) including Broca’s area, during execution, observation and imitation of verbal and nonverbal lip forms [41]. The stimuli were presented in the form of static images illustrating orofacial gestures that solely imply action (i.e., motionless). Interestingly, the results yielded strong BOLD signals evoked bilaterally in the MNS, including Brodmann’s areas 44/45 (Broca’s area), during pure perception of lip forms. This finding explicitly demonstrated that viewing visual orofacial stimuli is sufficient to trigger activity in the distributed language network, including areas involved in word-finding and speech production. We, therefore, hypothesized that providing SVC in the form of muted videos presenting lips articulating target words, might improve verbal performance, suggesting improved retrieval in participants with aphasia.

Second, we aimed to empirically explore the effects of SAC on lexical access and verbal execution in the same group. We chose to study whether semantically relevant sounds positively impact naming based on the notion of an embodied inference-driven language network, which proposes that auditory and conceptual brain systems are neuroanatomically and functionally coupled [42, 43], driven by the statistics of real-world interaction [34, 35]. Specifically, a functional Magnetic Resonance Imaging (fMRI) study [42] revealed that cortical activations induced by listening to sounds of objects and animals (e.g., “ringing” or “barking”) overlap with activations induced by merely reading words that contain auditory features (e.g., “telephone”, “dog”). The authors reported the overlap in the posterior superior temporal gyrus (pSTG) and middle temporal gyrus (MTG), which suggests that common neural sources underlie auditory perception and processing of words that comprise acoustic features. Critically, MTG plays a significant role within the brain’s language network during syntactic processing in both comprehension and production of speech [44]. For example, MTG was shown to subserve the retrieval, including selection and integration, of lexical–syntactic information in a syntactic ambiguity resolution task [45, 46]. Interestingly, pSTG is also involved in speech production, which is evidenced by clinical studies of conduction aphasia [47] as well as behavioral and imaging experiments with healthy subjects who performed tasks that included word generation [48], reading [49], syllable rehearsal [50], and naming [51–53]. Hence, we hypothesized that providing aphasia patients with SAC may facilitate naming possibly by activating brain regions involved in language production processing.

Similar to phonological and semantic cueing, we reasoned that, if the proposed SVC and SAC strategies are beneficial for the recovery of anomic disturbances in aphasia, they will foster naming accuracy and communication skills. We delivered and tested the efficacy of both types of cues in the context of longitudinal clinical intervention in which participants underwent a peer-to-peer specific Virtual Reality (VR)-based language therapy using the Rehabilitation Gaming System for aphasia (RGSa) [22], which is an evidenced-based system that incorporates principles of Intensive Language Action Therapy (ILAT) [54–56].

## Methods

### Participants

Ten participants with chronic (> 6 months post-stroke, mean (SD): 69.9(48.7)) aphasia participated in the study (age: Mean (SD): 57.6(9.9)). We included participants with moderate-to-severe stages of non-fluent aphasia as identified by a standard screening tool [57]. All participants were right-handed as assessed by the Edinburgh Handedness Inventory [58] and suffered a single left-hemispheric stroke affecting frontotemporal and parietal cortical areas, as evidenced by CT or MRI-scans. Participants were excluded if (1) they had a speech and language disorder caused by a neurological deficit other than stroke, (2) they had severe and untreated forms of cognitive disorders (assessed by the Mini-Mental State Examination [59]) and motor impairments (determined using Fugl-Meyer Assessment Upper Extremity [60]), which could adversely affect participation in the study and interaction with the proposed system, (3) if 2 years before the enrollment they participated in alternative intensive interventions, or (4) if they were currently using another computer program that trains naming or word finding. The demographic sample characteristics of all participants are presented in Table 1.

**Table 1:** Sociodemographic patient characteristics

ID	Age	Sex	Etiology	Chronicity (m)	Severity
9	58	M	Ischemia	6	Severe
10	39	F	Ischemia	83	Severe
11	64	M	Ischemia	46	Severe
12	63	F	Hemorrhage	72	severe
13	62	M	Ischemia	106	Severe
14	56	F	Ischemia	144	Severe
15	43	F	Hemorrhage	72	Severe
16	55	F	Ischemia	6	Moderate
17	75	M	Ischemia	144	Moderate
18	61	M	Ischemia	20	Moderate
**Mean (SD)**	57.6 (9.9)			69.9 (48.7)	

The reported paradigm deployed a within-subjects design. The experimental procedures followed written consents from all the involved participants. The study was further approved by the local Ethical Committee from the Hospital Universitari Joan XXIII and registered on ClinicalTrials.gov (NCT02928822) [22]. Clinical results of the randomized controlled trial are reported in [22].

### Treatment protocol and setting

All participants received five weekly intervention-sessions for 2 months. The duration of each session was 30-40 min. Thus, the full treatment included a total of approximately 23 h per participant.

The proposed cueing strategies were integrated into a novel language rehabilitation paradigm, the so-called Rehabilitation Gaming System for aphasia (RGSa) [22, 61]. Inspired by Intensive Language Action Therapy (ILAT) [54], RGSa is a VR-based rehabilitation tool administered in the form of an interactive language game that aims at practicing both speech production and comprehension by training frequent and behaviorally relevant communication acts such as making a request [22, 54, 62]. To this end, during therapeutic sessions of RGSa, ten participants were split into dyads (i.e., five pairs of two participants) and required to engage in a turn-based game played in a peer-to-peer setting without the involvement of a therapist [63].

The therapeutic setup of RGSa included two personal computers (Vaio, Japan) connected through a local area network, two headsets (EX-01 BluetoothR, Gioteck, Canada), and two motion tracking sensors (Kinect2, Microsoft, USA). Participants sat in a hospital ward in front of each other facing their respective screens which displayed the virtual environment from the first-person perspective. The virtual scene aimed to represent the actual setting. Thus, it consisted of two avatars seated at the respective sides of the table such that participants could see their virtual arms and the avatar of their interlocutor. On the virtual table, there was a set of three identical objects (see Stimuli) simultaneously available for selection. The movements of the real arms were continuously tracked by the Kinect and mapped in the real-time onto the arms of the virtual avatar. This method enabled interaction with the virtual world and, in particular, the virtual objects.

The objective of each session, and each participant was to collect as many objects as possible by requesting them from the other player or handing them over when required [22, 54]. At the beginning of each (daily) session, one of the participants from a dyad (e.g., PlayerA) was randomly assigned to initiate the game. Every trial consisted of the following three steps:

1. *PlayerA chooses the desired object.* PlayerA indicates the choice of the object for request by reaching towards it. To select the object, players were required to place the avatar’s hand over that object for three consecutive seconds. Once selected, to increase saliency and facilitate interaction, the object would light up in yellow and start rotating slowly over the vertical axis. Critically, to test our prediction about the beneficial effects of multisensory cueing on word production, we provided either SVC or SAC immediately after object selection to half of the stimuli (see Stimuli).
2. *PlayerA verbally requests the matching object from PlayerB.* After object selection, PlayerA had to utter a verbal request to obtain the matching object from the opponent. The use of politeness forms and full phrases (“Please, could you pass me the *pancake*”) was encouraged but not necessary.
3. *PlayerB reacts to the request.* In case the request was not understood, PlayerB had to ask PlayerA to repeat or clarify the request until it was clear. Whenever PlayerB understood what object was being requested, they were required to hand over the matching object to PlayerA by reaching towards it and holding the virtual hand over it for three consecutive seconds.

**Fig. 1:**
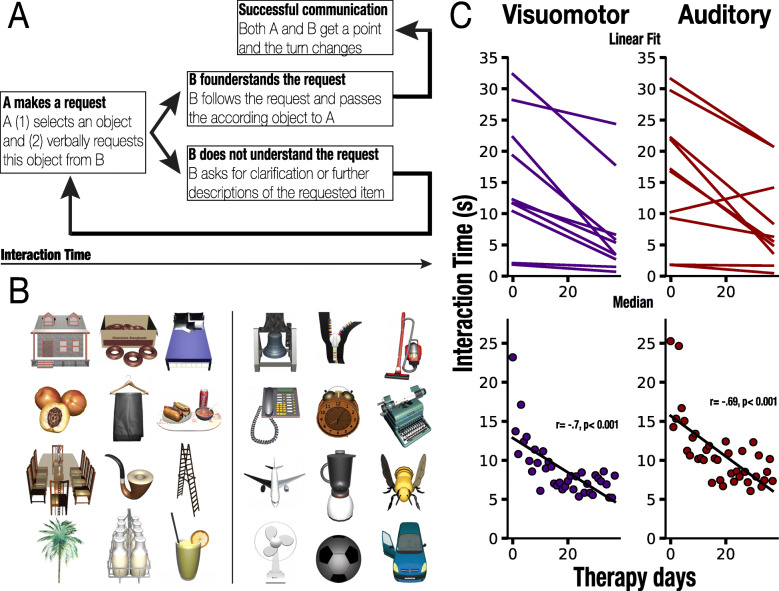
**a** Illustration of the Interaction Time (IT) measure, possible moves, and speech- acts. **b** Example of the materials. Left: stimuli undergoing SAC, right: stimuli undergoing SVC. **c** Fit for each participant’s averaged IT over the therapy interval for all the stimuli undergoing Silent Visuomotor (SVC, violet) and Semantic Auditory (SAC, red) cueing. Upper panels: Lines represent linear regression models for individual participants including cued and non-cued trials. Lower panels: Median ITs of all the participants including all stimuli for each therapy session

The completion of these three steps comprised a successful communicative interaction, which included both a successful speech act (performed by PlayerA) and successful comprehension (performed by PlayerB). After such a sequence of events, both participants saw the two matching objects on the screen (i.e., positive feedback), heard the correct pronunciation of the target word through headphones (i.e., reinforcement), received a point, and the turn changed (Fig. 1a). After a short delay, a new pseudo-randomly chosen object was generated and spawned for both players such that there were always three objects on the table. The goal for each participant, and each session was to request and collect a total number of 36 objects. Consequently, the RGSa session ended when participants completed this task which usually took approx. 30–40 min. The system continuously stored the moves of both participants as well as the game events. Finally, a previously trained therapy assistant supervised all the sessions. Their role was to monitor the participants during the intervention interval and support them when a trial could not be realized independently. Critically, the assistant did not offer any elements of standard speech and language therapy. The detailed methodology of the RGSa treatment is available in [22].

### Stimuli and multisensory cueing

The stimuli used in the study consisted of 120 items presented in the form of three-dimensional virtual objects (Fig. 1b). It has been widely accepted that properties of target stimuli, including visual complexity, name agreement as well as imageability, might affect picture identification and, consequently, lexical retrieval [18, 64–66]. Hence, to ensure visual unambiguity, all objects were first evaluated by healthy participants and clinicians involved in the trial. Furthermore, all items were categorized regarding frequency, semantic category, complexity, and phonemic similarity, and they were matched for the syllable length and semantic category.

To test our hypotheses, the one hundred twenty stimuli were classified into two categories, including (A) sixty items without semantically related such as “pancake” and (B) sixty items for which acoustic features are highly relevant such as “telephone.” The first group of stimuli (i.e., A) underwent the Silent Visuomotor Cueing (SVC) [37]. The cues consisted of displaying videos that showed recordings of a speech and language therapist who articulated each of the sixty stimuli following the criteria of standard phonological cueing [10, 11]. Importantly, however, for this study, instead of the initial phoneme/s, the therapist pronounced full target words, and the recorded voice was muted such that the cues were silent. Every video depicted a part of the face of the therapist, including mouth and nose. The videos were recorded in the Clinica de l’Hospital Universitari Joan XXIII de Tarragona, Spain. The second group of stimuli (i.e., B) underwent the so-called Semantic Auditory Cueing (SAC). SACs consisted of providing a sound that is semantically relevant to the object selected for the request, for example, the sound of ringing for the object representing “telephone,” or the sound of an engine revving up for the object representing “car.”

For each pair of participants, the stimuli were delivered in a pseudorandomized order, counterbalanced within each week. Cueing strategies were provided to half of the practiced stimuli. Specifically, for each participant, SVCs were delivered in 50% of the items without acoustic features (group A), and SACs were delivered in 50% of the items with semantically relevant sound (group B). In both cases, the cues were provided immediately after object selection, once per trial. All participants were given a wireless headset through which they heard feedback from the system.

### Measures

To evaluate the naming accuracy of the target stimuli, we administered the primary outcome measure, in particular, the Vocabulary Test (VocabT), which included all the trained items [22]. For each word, participants could score a maximum of 5 points (0: no verbal utterance, 1: utterance followed by full phonetic priming, 2: utterance followed by priming of the initial phoneme, 3: utterance followed by full silent orofacial hint, 4: utterance followed by a silent orofacial hint of the first phoneme, 5: utterance followed by no hint). The test was administered six times over the intervention period to determine the baseline (week 0), changes in accuracy at weeks 2, 4, 6, 8, as well as the follow-up period at week 16.

As the secondary outcome measure, we computed Interaction Times (ITs, see Fig. 1a) for all stimuli and therapy sessions. IT was an objective quantification of improvement in communicative effectiveness which captured the time of successful goal-oriented peer-peer interaction. Hence, we defined IT as the time interval between the selection of the target object for the request and the collection of the matching object from the opponent. Consequently, each IT included lexical access, articulation of the request, comprehension of the target word, and the motor response of the opponent. All pairs of participants remained the same during the therapy interval, which ensured that the times of motor responses were constant, thus not influencing the language-related results.

### Data analysis

We used the Wilcoxon signed-rank test to evaluate within-groups changes and Mann-Whitney U-test for between-groups comparisons. All comparative analyses used two-tailed tests and a standard level of significance (*p* < .05).

## Results

We aimed to determine the effects of SVC and SAC on naming and communication in individuals with chronic non-fluent aphasia.

**Fig. 2:**
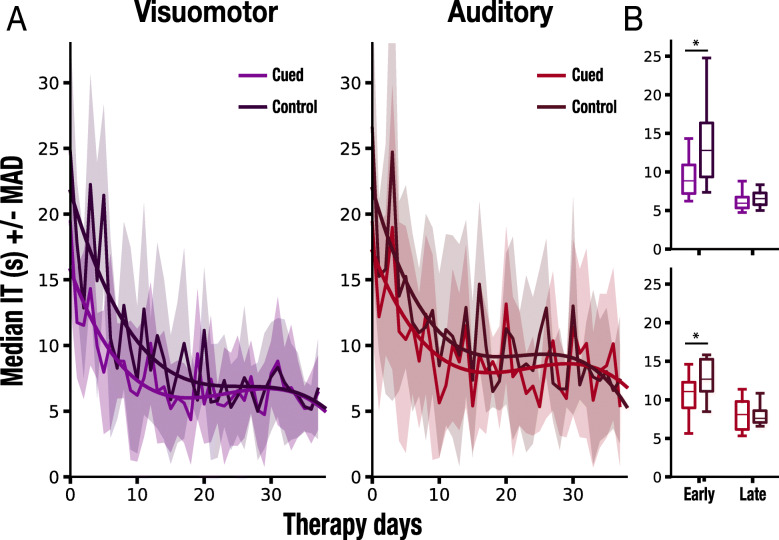
**a** Evolution of median ITs for cued on non-cued stimuli over the therapy sessions. Lines represent nonlinear regression models for cued and non-cued visuomotor (violet) and auditory (red) cues. **b** Quantification of differences in ITs for SVC and SAC between cued and non-cued stimuli in the early (first 15) and late (last 15) therapy sessions

First, we evaluated naming accuracy as measured by a standard clinical scale VocabT. Our results yielded significant improvement on the proposed scale from baseline at each evaluation point including week 2 (W2, *p* = 0.01), 4 (W4, *p* = 0.006), 6 (W6, *p* = 0.005), 8 (W8, *p* = 0.005), and the follow up (W16 *p* = 0.005) (see Table 2). Second, we computed the change in Interaction Times (ITs). The analysis of the evolution of the ITs throughout the intervention interval (40 days) yielded a significant decrease for all the presented stimuli (*N* = 120), including cued and non-cued stimuli (*r =* −.61, *p* < 0.001). Critically, we also found significant improvements in the ITs measure for the two subsets chosen to undergo SVC (*N =* 60, Fig. 1c Left, *r =* −.7, *p <* 0.001) and SAC (*N =* 60 Fig. 1b Right, *r =* −.69, *p <* 0.001), respectively (Fig. 1c Left panels and Fig. 1c Right panels), when accounting for all cued and non-cued trials. Subsequently, to estimate the effects of the two types of multisensory cues on verbal expression, we compared the ITs between cued and non-cued stimuli for all the intervention days as well as for the early and late trials (Fig. 2a). A Wilcoxon signed-rank test demonstrated a significant difference between all cued and non-cued stimuli in SVC (*p* = .001) and SAC (*p* = .003). Specifically, we found that the difference between cued and non-cued trials was statistically significant during the early therapy sessions (*N* = 15) both for SVC (*p* = .002) and SAC (*p* = .001) (Fig. 2b Upper panel). No differences in ITs were found in the late sessions for neither SVC (*p* = .73) or SAC (*p* = .53) (Fig. 2b Lower panel). Moreover, the analysis yielded no differences in ITs between non-cued SAC and SVC stimuli in the early sessions (*p* = .28) establishing that the chosen subsets did not differ regarding difficulty.

**Table 2:** Outcome measures at weeks 2, 4, 6, 8, and 16 (followup). Bold values indicate significant differences (*p* < .05). *P*-values for within-group analysis were obtained with Wilcoxon signedrank test

Within-group analysis Mean (SD) - Median 95 % confidence interval for the mean (lower and upper bound)		
W2	δ(W2-BL)	*p-value*
86.93(10.26)-88.60	7.8(6.85)-10.73	**.01**
[79.19-94.67]	[2.63-12.97]	
W4	δ(W4−BL)	
90.01(9.81)-94.09	10.88(7.03)-12.54	**.006**
[82.61-97.41]	[5.57-16.19]	
W6	δ(W6−BL)	
92.47(10.63)-97.37	13.34(7.33)-13.19	**.005**
[84.45-100.49]	[7.81-18.87]	
W8	δ(TW8−BL)	
95.06(8.31)-98.44	15.93(7.56)-13.68	**.005**
[88.79-101.33]	[10.22-21.64]	
W16	δ(W16−BL)	
94.78(8.79)-98.77	15.65(7.02)-14.42	**.005**
[88.15-101.41]	[10.35-20.95]

Finally, we evaluated whether changes in communicative effectiveness as measured by the ITs reflect the improvement in verbal production. To this aim, we examined the relationship between the proposed measure automatically stored by the system (IT), and naming accuracy as quantified using the clinical scale (VocabT) which showed a significant increase from baseline after the intervention (Wilcoxon signed-rank: *p* = .007) [22]. For the analysis, we extracted ITs including all cued and non-cued stimuli from all the therapy sessions for each participant and computed mean ITs collected on the date of the administration of the VocabT ±1 day. Spearman’s correlation revealed a significant relationship between the mean ITs and the VocabT scores across participants (*r* = −.89, *p* = −.03), suggesting that ITs may be regarded as a relevant measure of verbal execution in participants with chronic non-fluent aphasia.

## Discussion

While phonological and semantic priming has been widely established [14–18], to the best of our knowledge, no study has explicitly explored the effects of silent visuomotor (SVC) and semantic auditory (SAC) cues on naming in people with aphasia who display anomia. Hence, in this study, we aimed to examine the effects of the proposed multisensory priming on accuracy and communicative effectiveness for a large set of items in ten participants with stroke-induced non-fluent aphasia at the chronic stage. To this aim, we used a VR-based language-rehabilitation protocol, the RGSa [22], in which dyads of patients practiced communicative acts (i.e., making a request) in the form of a turned-based game. We administered SVCs and SACs in a pseudorandomized manner at the moment when the active player selected the object to be requested from the interlocutor. Naming accuracy for the trained stimuli was evaluated five times during the intervention and once at the follow-up period using a standard clinical scale. Moreover, the RGSa system allowed for an objective, automatic, and continuous quantification of the priming effects on communicative effectiveness [22]. In particular, for each participant and all the therapeutic sessions, we stored and computed the so-called Interaction Times (IT, Fig. 1a), which indicated the interval from object selection for the request to the reception of its matching counterpart from the opponent. On the one hand, we hypothesized that naming accuracy for the trained stimuli and communication effectiveness would improve through the intervention sessions of RGSa as reflected by an increase of scores on VocabT and decrease of ITs, respectively. On the other hand, we predicted that, if the proposed SVC and SAC facilitate lexical access, they may result in faster ITs as compared to the non-cued stimuli.

First, the analysis of the vocabulary test revealed that the participants significantly improved naming accuracy for both cued and non-cued stimuli at each time step compared to baseline. Among other positive clinical outcomes reported in [22], the changes on the VocabT demonstrate that the RGSa intervention had beneficial effects on the recovery of naming and, critically, the retention of the acquired changes as evidenced by the follow-up assessment. We believe that peer-peer interactions of RGSa sessions whereby participants were required to use every day-like language might have positively influenced the frequency of communication in social situations outside of the hospital, thus reinforcing language use and improving the naming accuracy of the trained vocabulary [22, 67, 68].

Second, to objectively quantify the improvement in communication, we used ITs that were stored by the RGSa system and computed automatically for each session and each subject without the therapist’s supervision. The ITs were designed to reflect successful interactions whereby the reward was reflected by an achievement of a behavioral goal (i.e., obtaining the requested item) rather than accurate naming per se. To this end, ITs were stored from the moment when the active player selected the desired object for the request until they received the corresponding object from the opponent. In line with our first hypothesis, the analyses of repeated measurements statistics, including both cued and non-cued trials, yielded a significant decrease in ITs over the therapy interval. Critically, we found that ITs were strongly and negatively correlated with the performance on the VocabT such that the less time it took the participants to request and receive the desired objects, the higher was their naming accuracy. This finding suggests that ITs reflect both general communicative effectiveness and, implicitly, naming fluency captured by a clinical scale. Having established the utility of this this new implicit IT measure of naming performance, we will now validate and integrate it a broader range of standardized outcome measures that have validated sensitivity to treatment-induced changes in aphasia rehabilitation [69].

Third, and most important, the central objective of this study was to determine the effects of SVC and SAC on naming and communicative effectiveness in people with aphasia. To quantify those effects, we compared the ITs between those trials in which the cues were provided at the moment of object selection (i.e., cued trials) and those when they were absent (i.e., non-cued trials). The results revealed differences in ITs between cued and non-cued stimuli for both SVC and SAC. Critically, these effects were significant, especially in the early intervention days, when the exposure to the target lexicon was still infrequent. No such differences were found in the late sessions, possibly because, at that stage, the acquisition of the target stimuli reached a plateau. We propose that the reported differences between cued and non-cued trials support the notion that both visuomotor (SVC) and acoustic (SAC) information indeed aids naming of the trained stimuli in patients with non-fluent aphasia even at the chronic stage of the disease, which is in line with the inference-based network perspective on naming [33, 43, 70]. One could argue, however, that the IT measure presents some limitations. Specifically, to capture successful interactions between two interlocutors, each IT comprised a set of actions performed by the active player, who requested the desired object, and their opponent. In particular, those actions included word retrieval and articulation of the request, on the one hand, and comprehension of the target word and motor response of the opponent, on the other. Since all of these actions could have potentially improved over the therapy sessions, changes in ITs could be reflecting changes in one, some, or all of the captured acts, which would constitute a confounding factor. To support the proposed interpretation that the reported ITs results demonstrate changes in naming rather than, e.g., motor performance, we observe that, first, as discussed above, our analysis yielded a strong and statistically significant relationship between ITs and the VocabT, which supports the hypothesis that, implicitly, ITs reflect naming accuracy. Second, since the multisensory cues were only provided to the active player at the moment of object selection, these cues could not have impacted either the comprehension of the speech act or the opponent’s motor responses. The significant difference between cued and non-cued trials, acquired independent of the fact that each IT included the interlocutor’s motor responses that may improve due to a non-specific practice effect, further supports the robustness of the IT measure. To account for variables that could impact ITs, including practice effects, we are currently enhancing automatic kinematic data analyses (e.g., movement trajectories, reaction and response times). Moreover, in follow-up studies we will analyse (1) the generalization of the reported effects and (2) the changes on the VocabT depending on whether the cues are present. Although the current experimental design, whereby each pair of participants were delivered the cues in a different pseudorandomized order, did not allow us to perform such analysis, we expect to see a more pronounced effect on the IT.

From a technological perspective, it is noteworthy that both the proposed multisensory cueing strategies and the IT measure could be transferred into real-world applications for individuals with language deficits, without requiring the assistance or supervision of a therapist. Specifically, they could be implemented into computer-based, wearable, or mobile technology as (1) a therapeutic strategy that facilitates naming, on the one hand, and (2) a diagnostic tool for changes in word production, on the other. The proposed methods could be extended by integrating an additional tool to measure improvement in individuals with aphasia. For instance, it could include an automatic measure of response times by subtracting the time of the actual verbal utterance from the moment of the selection of the target word. We did not implement such technology in the current study for two reasons. First, it would require a speech recognition system, which is not suitable for our sample that includes participants with moderate-to-severe stages of aphasia. Second, this method would allow for the quantification of naming speed rather than communicative effectiveness whereby the primary objective is to achieve the behavioral goal by obtaining a desired object from the interlocutor. However, we believe that a combined approach, including the assessment of goal-directed dyadic interaction and the naming speed, would be ideal, informing the users about possible changes in specific stages of naming [4, 5].

Of fundamental clinical relevance, the reported results provide evidence for the beneficial effects of multisensory cueing on verbal execution. This suggests that integrating SVC and SAC in the rehabilitation of aphasia could foster language-production skills within and outside of the clinic and even at the chronic stages of the disease. Furthermore, these findings might find applications as predictors of post-stroke aphasia recovery. Specifically, there is both behavioral and neuroimaging evidence which demonstrates that the responsiveness to cues (i.e., classical phonological cueing) predicts immediate treatment outcomes in other phonological treatment approaches [28, 71]. We designed SVCs such that they contain visuomotor information related to the phonology of a target word while SACs contain the auditory information related to the semantics of a target word. Future studies should evaluate if responsiveness to SVC and SAC is predictive of outcomes on phonological and semantic tasks, respectively.

Of scientific relevance, our findings are consistent with sensorimotor accounts of language processing [33, 43, 70] highlighting the relevant coupling between brain networks underlying perceptual and motor brain regions. They also provide supporting evidence for network interpretation of speech production whereby different stages of naming are governed by principles of statistical inference across all available perceptual sources [33]. On the one hand, the beneficial effects of SVCs in the early intervention sessions might be attributed to increased activity of the language networks related to the processing of orofacial gestures, thus facilitating articulation [40, 41]. On the other hand, SACs might have facilitated word production by activating semantic regions, including pSTG and MTG, thus facilitating lexical access and consequently naming [42]. Future studies shall systematically investigate the neurophysiological underpinnings of both types of cues.

## Conclusions

This study extends current empirical and clinical framework on language rehabilitation by showing the efficacy of multisensory cueing in fostering naming even at the chronic stages of aphasia [15, 16, 18, 20]. Critically, the proposed strategies may be easily and at a low cost integrated into digital technology that may be used after hospital discharge to improve the quality of life of the patients. Finally, our findings support the hypothesis of the inference-based network at the basis of language production [33].

## Data Availability

The data that support the findings of this study are available from the corresponding author upon request.

## Abbreviations

SVC: Silent Visuomotor Cueing
SAC: Semantic Auditory Cueing
ILAT: Intensive Language Action Therapy
VR: Virtual Reality
RGSa: The Rehabilitation Gaming System for aphasia
VocabT: Vocabulary Test
IT: Interaction Time
pSTG: Posterior superior temporal gyrus
MTG: Middle temporal gyrus
